# Histone Deacetylase Inhibitor Trichostatin A Reduces Endothelial Cell Proliferation by Suppressing STAT5A-Related Gene Transcription

**DOI:** 10.3389/fonc.2021.746266

**Published:** 2021-09-23

**Authors:** Yize Li, Yongmei Zhao, Hongyan Peng, Jing Zhang, Lun Bo, Lei Wen, Wenchao Liu, Wendong Bai, Hongmei Zhang

**Affiliations:** ^1^ Department of Clinical Oncology, Xijing Hospital, Fourth Military Medical University, Xi’an, China; ^2^ Department of Hematology, Xinjiang Command General Hospital of Chinese People’s Liberation Army, Urumqi, China; ^3^ Department of Internal Medicine, 63650 Military Hospital, Urumqi, China; ^4^ Department of Endocrinology, Xijing Hospital, Fourth Military Medical University, Xi’an, China; ^5^ Department of Clinical Laboratory Center, Xinjiang Command General Hospital of Chinese People’s Liberation Army, Urumqi, China

**Keywords:** HDAC, HDAC inhibitors, angiogenesis, HUVEC, STAT5A

## Abstract

Inhibitors of histone deacetylases (HDACi) have shown promising effects in preclinical applications for the treatment of many diseases. Confusedly though, the effects of the HDACi trichostatin A (TSA) on angiogenesis are variable among different diseases. This study investigated the direct effects of TSA on endothelial cells, which plays essential roles in angiogenesis and the underlying molecular events. TSA reduced the viability of human umbilical vein endothelial cells (HUVECs), in which proliferation-related genes including *BIRC5*, *CKS1B*, and *NDC80* were found to be involved. Furthermore, signal transducer and activator of transcription 5 A (STAT5A) was demonstrated to be reduced by TSA and to mediate TSA-induced downregulation of *BIRC5*, *CKS1B*, and *NDC80* and HUVEC proliferation. Mechanistically, data showed that STAT5A directly bound to the promoters of *BIRC5*, *CKS1B*, and *NDC80* and activated their transcription through special DNA sequence sites. Finally, the TSA–STAT5A–*BIRC5*, *CKS1B*, and *NDC80* axis also worked in a cancerous endothelial cell angiogenesis model. The results of this study revealed novel mechanisms underlying the effects of TSA on endothelial cells and provided insights for angiogenesis-associated diseases.

## Introduction

Angiogenesis is the physiological process of new blood vessel formation by endothelial cells. Many different growth factors, including vascular endothelial growth factor (VEGF), basic fibroblast growth factor (bFGF), and matrix metalloproteinases (MMPs), are known to promote angiogenesis by endothelial cells (1). Under normal conditions, angiogenesis plays an important role in a wide range of physiological processes, such as wound healing and fetal development. Under pathological conditions, it contributes to many angiogenic diseases and tumorigenesis. Thus, elucidating the mechanisms of angiogenesis in both health and disease and developing drugs that can regulate angiogenic processes are matters of great clinical significance.

Histone acetylation is recognized as a fundamental process in the regulation of gene expression and an important epigenetic modification associated with the induction of physiological and pathological processes. Histone deacetylases (HDACs) determine the status of histone acetylation, and inhibitors of histone deacetylases (HDACi) have emerged as potential therapeutic agents that can reverse aberrant epigenetic changes associated with cancer and cancer-related angiogenesis (2). Several classes of HDACi have been shown to possess potent and specific activities for the treatment of specific diseases in preclinical studies (3). For example, the HDACi trichostatin A (TSA; obtained from *Streptomyces hygroscopicus*) is an antifungal antibiotic and a selective inhibitor of class I and II HDACs (4). TSA can inhibit cell cycle progression and regulate gene expression through the removal of acetyl groups from histones to alter the access of transcriptional factors to genomic DNA. Interestingly, TSA has opposing effects on angiogenesis in different disease models. For example, TSA enhances angiogenesis in a model of myocardial repair, but inhibits angiogenesis in several models of cancer progression (5).

Indeed, the direct effects of HDACi on endothelial cells as well as the underlying mechanisms remain largely unknown. Therefore, the present study aimed to understand how TSA affects endothelial cells and elucidate the responsible mechanisms. We first analyzed the NCBI gene expression omnibus (GEO) dataset #GSE5856 to identify differentially expressed genes in human umbilical vein endothelial cells (HUVECs) treated with TSA. We then observed the effects of TSA treatment on HUVEC viability and the expression of the identified genes and further investigated the underlying mechanisms. Finally, we investigated the effects of TSA on tube formation in two cancer endothelial cell models. The results obtained in this study provide important insight into the genes involved in the effects of TSA on endothelial cells.

## Materials and Methods

### Materials

TSA was obtained from Selleckchem (Houston, TX, USA). TSA was dissolved in 70% ethanol to prepare a stock concentration of 500 nM, which was then stored at −20°C until use. Anti-baculoviral IAP repeat containing 5 (*BIRC5*) (cat #ab469), anti-cyclin-dependent kinases regulatory subunit 1 (*CKS1B*) (cat #ab72639), anti-*NDC80* (cat #ab186839) antibodies, and anti-STAT5A (cat #ab32043) antibodies were obtained from Abcam (Cambridge, UK); anti-STAT5A (cat #94205, ChIP grade) was obtained from Cell Signaling Technology (Danvers, MA, USA); and a mouse monoclonal anti-β-actin antibody (cat #A5441) was obtained from Sigma-Aldrich Chemicals (St. Louis, MO, USA).

### Patient Samples, Cell Lines, and Treatments

This study was registered with and approved by the Ethics Committee of the First Affiliated Hospital of Fourth Military Medical University (registration number: KY20213431-1). Primary ovarian carcinoma tissues, breast carcinoma tissues, and adjacent normal tissues were collected from patients in Xijing Hospital. HUVECs were obtained from the American Type Culture Collection (ATCC, Manassas, VA, USA). HUVECs were maintained in EGM-2 endothelial cell growth medium (Lonza, Basel, Switzerland) at 37°C in a humidified incubator with 5% CO_2_ and 95% O_2_. For drug treatment, cells were grown to 70%–80% confluency and then treated with different doses of TSA for specific durations of time. The human ovarian carcinoma cell line SKOV3 and the human breast carcinoma cell line MCF-7 were purchased from the ATCC and maintained in DMEM supplemented with 10% FBS (6, 7).

### Bioinformatics Analysis

Microarray data for differentially expressed genes in HUVECs treated with or without TSA were downloaded from the NCBI GEO database with the accession number GSE5856 (https://www.ncbi.nlm.nih.gov/geo/query/acc.cgi?acc=GSE5856). In particular, in differentially expressed genes (DEGs), we considered the false discovery rate (FDR) <0.05 and a log_2_ fold change |log_2_FC| >1. Based on the list of 214 DEGs, we performed gene ontology enrichment analysis of DEGs using the DAVID bioinformatics resources website (https://david.ncifcrf.gov/) (8). The ClueGO+CluePedia tool in Cytoscape was used to perform cluster-based analysis of the biological processes dysregulated in TSA-treated HUVECs (**Supplementary Figure 2**). The structure of STAT5A was predicted on the Swiss-model website (https://swissmodel.expasy.org). Furthermore, we predicted the signal transducer and activator of transcription 5 A (STAT5A)-binding sites [gamma-activated site (GAS) 5′-TTCNNNGAA-3′] of the *BIRC5*, *CKS1B*, and *NDC80* promoter regions using a tool from the NCBI website (9). The data were uploaded in github website (https://github.com/bwddcgzl/EC-STAT5A.git).

### Isolation of Endothelial Cells

For isolation of endothelial cells from human ovarian carcinoma tissue, breast carcinoma tissue, and adjacent normal tissues, tissues were cut into pieces with a surgical knife and digested with collagenase 1 (Gibco, 17100017) in DMEM for 2 h at 37°C. The solution derived from the tissue was filtered, and red blood cells were eliminated using red blood cell lysis buffer. A single-cell suspension was prepared and resuspended to achieve a cell concentration of 1 × 10^8^ cells/ml. For labeling with Dynabeads (Invitrogen, 11155D), the beads were suspended in a vial, washed *via* the placement and removal of a magnet, and then resuspended. The prepared cell solutions were incubated with the resuspended Dynabeads, before washing as described to obtain the bead-bound CD31+ endothelial cells. Pellets of these cells were transferred to the preferred cell medium (10).

### Tumor-Conditioned Medium Preparation

Cells of the human breast carcinoma cell line MCF-7 and ovarian carcinoma cell line SKOV3 were first seeded in a 10-cm dish in growth medium containing 10% FBS, separately. To prepare the tumor-conditioned medium (TCM), the growth medium was replaced with serum-free medium, and the breast and ovarian cancer cells were incubated for an additional 48 h. The TCM was collected and filtered through a 0.2-μm filter for use in culture experiments.

### Quantitative Real-Time Polymerase Chain Reaction

Total RNA was isolated from the HUVECs using TRIzol reagent (Invitrogen) and reverse transcribed into cDNA using the miScript Reverse Transcription Kit (Qiagen, Valencia, CA, USA) according to the instructions of the manufacturer. Quantitative real-time polymerase chain reaction (qRT-PCR) amplification was then performed with primers of human *BIRC5*, *CKS1B*, *NDC80*, and STAT5A and a housekeeping gene *GAPDH* (GenePharma, Shanghai, China). The primers were designed using the Premier 5.0 software (Biosoft International, Palo Alto, CA, USA; see **Supplementary Table 1**). The qRT-PCR conditions were set to 95°C for an initial 5-min incubation, then 35 cycles of 95°C for 10 s, 58°C for 30 s, and 72°C for 30 s, followed by a final 10-min incubation at 72°C. The samples were then stored at 4°C. GAPDH mRNA was used as an internal loading control. Relative mRNA levels were calculated using the 2^−[Δ][Δ]^Ct method.

### Cell Proliferation Assay

HUVECs in the logarithmic growth phase were seeded into a 96-well plate at a density of 2,000 cells per well and grown overnight. The cells were then treated with different doses of TSA for up to 5 days, and the cell numbers were calculated daily for 5 days using a Cellomics ArrayScan VTI reader (Cellomics, Inc., Pittsburgh, PA, USA) with analysis settings adjusted according to the input parameters. The experiments were performed in triplicate and repeated three times independently.

### Plasmid Construction and Gene Transfection

The STAT5A open reading frame (ORF) cDNA was amplified by PCR from HUVECs and subcloned into pcDNA3.0 (Invitrogen), while plasmids carrying human *BIRC5*, *CKS1B*, and *NDC80* cDNA and promoter regions were amplified as described previously (7, 11). Plasmids carrying a mutated GAS site (5′-TTCNNNGAA-3′) within the distal *BIRC5*, *CKS1B*, and *NDC80* promoter regions were constructed using the site-directed mutagenesis kit from Stratagene (La Jolla, CA, USA) as described previously (12). After confirmation of the DNA sequences, HUVECs were transfected with these plasmids. The PCR primers are listed in the **Supplementary Table 1**. Furthermore, we chemically synthesized small interfering RNAs (siRNAs) and control RNAs and used them to knock down STAT5A, *BIRC5*, *CKS1B*, and *NDC80* expression to study the effects on HUVEC proliferation. For transfection, HUVECs were seeded into six-well plates at a density of 2.5 × 10^5^ cells per well, grown for 24 h, and then transfected with plasmids using Lipofectamine 2000 (Invitrogen) for 48 h according to the protocol of the manufacturer. The cells were then subjected to the described assays. GAS site wild-type and mutation sequence was uploaded in github website (https://github.com/bwddcgzl/EC-STAT5A.git).

### Chromatin Immunoprecipitation Assay

A chromatin immunoprecipitation (ChIP) assay was performed to assess *in vivo* DNA–protein interactions at the *BIRC5*, *CKS1B*, and *NDC80* promoters using the ChIP-IT express enzymatic kit in accordance with the instructions of the manufacturer (Active Motif, CA, USA). Briefly, cells were grown, treated with TSA, and then cross-linked with 1% formaldehyde at room temperature for 10 min to induce the cross-linking of protein–DNA complexes. After that, cells were washed twice with ice-cold phosphate-buffered saline (PBS) containing protease and lysed using the ChIP lysis buffer containing cell lysis buffer containing protease inhibitor cocktail II. Cells were centrifuged at 4°C for 5 min at 800×*g*, and the supernatant was removed. Then, cell pellets were resuspended in nuclear lysis buffer containing protease inhibitor cocktail II. After sonicating on wet ice, cell lysates were centrifuged, and the supernatant was collected in fresh microfuge tubes. Dilution buffer was added into each tube containing 50 μl of chromatin. Then, 5 μl (1%) of the supernatant was collected for future use as the “input.” The immunoprecipitating antibody and 20 μl of fully resuspended protein A/G magnetic beads were incubated (with rotation) with the STAT5A antibody at 4°C overnight with end-over-end rotation. Samples were washed with wash buffer and eluted with SDS elution buffer. Following elution, samples were reverse cross-linked at 65°C for 4 h, treated with RNase A at 37°C for 2 h, and mixed with CaCl_2_ and proteinase K at 37°C overnight. The immunoprecipitated DNA samples were isolated and used as templates for PCR amplification of *BIRC5*, *CKS1B*, and *NDC80* promoters or the target region of the predicted GAS sites. The primers are listed in the **Supplementary Table 1**. The PCR conditions were set to an initial 5-min incubation at 98°C followed by 32 cycles of 94°C for 30 s, 56°C–58°C (depending on the primer) for 30 s, and 72°C for 30 s, with a final 10-min incubation at 72°C, as described previously (13).

### Western Blotting Analysis

Total cellular protein was extracted from HUVECs using a radioimmunoprecipitation assay buffer and quantified. Samples of 50 μg protein were separated by 10% SDS-polyacrylamide gel electrophoresis and transferred onto polyvinylidene difluoride (PVDF) membranes. The membranes were then blocked in non-fat dry milk–PBS solution at room temperature for 1 h and incubated with primary antibody at a dilution of 1:800 at 4°C overnight. The next day, the membranes were washed with PBS-Tween 20 (PBS-T) three times briefly and then incubated with an appropriate anti-rabbit or mouse IgG conjugated with horseradish peroxidase (HRP) at a dilution of 1:2,000 for 1 h at room temperature in darkness. The membranes were subsequently incubated briefly with an enhanced chemiluminescence (ECL) solution (Amersham Biosciences, NJ, USA). β-Actin was used as a loading control by stripping the membranes and reblotting with an anti-β-actin antibody.

### Luciferase Reporter Assay

The luciferase reporter assay was performed in 48-well plates as described previously (14) with the protocol of the manufacturer using the dual luciferase assay kit from Promega (Madison, WI, USA). HUVEC cells were grown and co-transfected with 100 ng of a firefly luciferase reporter plasmid pGL3 carrying different wild-type or mutant constructs or the Renilla luciferase vector pRL-TK (5 ng; for normalization) from Promega using Lipofectamine 2000 transfection reagent (Invitrogen, Carlsbad, USA) according to the protocol of the manufacturer. After 48 h, the total cellular protein was extracted using a radioimmunoprecipitation assay buffer, quantified, and subjected to the luciferase reporter assay. Luciferase activity was determined by calculating the ratio of firefly/Renilla luciferase activity and recorded using GloMax 96 microplate luminometer (Promega, USA). The experiments were performed in triplicate.

### Tube Formation Assay

HUVECs transfected with the specified plasmids were cultured in TCM and treated with or without TSA for 48 h. The cells were then seeded in 24-well plates coated with Matrigel (BD Biosciences, NJ, USA). After incubation at 37°C with 5% CO_2_ for 24 h, five randomly selected fields in each well were photographed using a Nikon inverted microscope. Total tube length and branch number were determined using ImageJ image analysis software (NIH, Bethesda, MD, USA) (15).

### Statistical Analysis

All numerical data are expressed as mean ± SD of at least three independent replicates and were statistically analyzed using PRISM, version 9 (GraphPad Software, CA, USA). Data were two-tailed Student’s *t*-test for qRT-PCR, luciferase assay, cell proliferation assay, and tube formation assay. A *P*-value equal to or less than 0.05 was considered statistically significant.

## Results

### TSA Significantly Decreased HUVEC Proliferation

**Figure 1:**
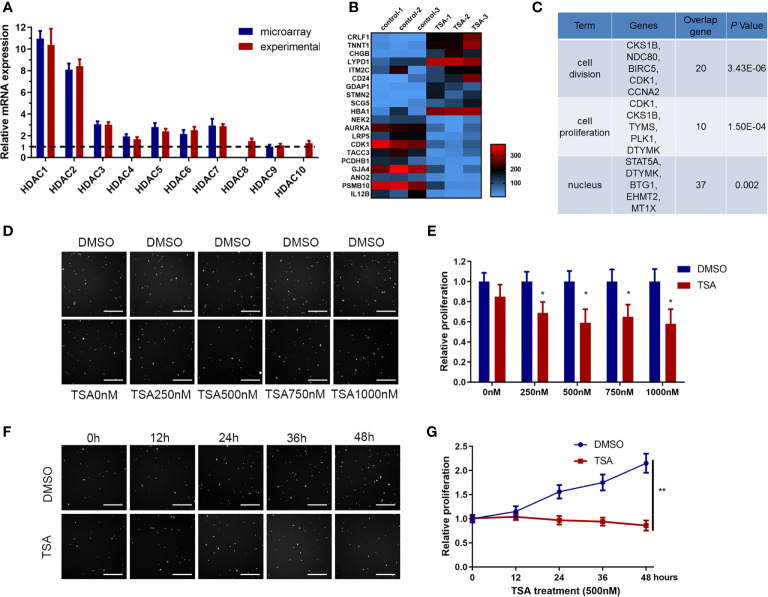
Trichostatin A (TSA) reduced human umbilical vein endothelial cell (HUVEC) proliferation. **(A)** Differential expression of HDAC genes from the GEO database data (#GSE5856) was tested by qRT-PCR. The relative value of the lower HDAC9 level was set to 1.0 as reference. **(B)** mRNA expression profiling of HUVECs with TSA treatment. HUVECs were grown and treated with TSA (500 nM for 24 h). Total RNAs isolated from experimental and control groups were subjected to microarray analysis. Heatmap was employed to visualize the top 20 genes. Shades of red represent increased gene expression, whereas shades of blue represent decreased expression. **(C)** Gene ontology enrichment performed by DAVID for gene expression profiling. **(D)** HUVECs were treated with TSA or DMSO at different concentrations for 24 (h) Cell proliferation of HUVECs was analyzed by Cellomics ArrayScan VTI assay. Scale bar 400 μm. **(E)** Quantification of the data from **(D)**. **(F)** HUVECs were treated with 500 nM TSA or DMSO for different durations of time. Cell proliferation of HUVECs was measured with Cellomics ArrayScan VTI assay. Scale bar 400 μm. **(G)** Quantification of the data from **(F)**. The bars represent means ± SD from three independent experiments. *P < 0.05, **P < 0.01 *vs*. the control group.

The HDACi TSA has been reported to influence angiogenesis, but its effects vary in different disease models. Elucidating how TSA directly affects endothelial cells and the underlying mechanisms is important for its future clinical application. First, we screened potential HDAC genes using data from a previous genome-wide microarray analysis of human HUVECs (GEO accession number: #GSE5856) and then confirmed the findings *via* qRT-PCR. The data showed that HDAC1–10 genes were coexpressed, with HDAC1 and HDAC2 as the predominant transcripts (**Figure 1A**). Then, we analyzed the HDAC transcripts in HUVECs after TSA treatment within the microarray data (#GSE5856) and identified the top 214 differentially expressed genes (**Figure 1B**). Analysis of the differentially expressed genes using the online software Cytoscape indicated that most TSA-regulated genes were enriched in endothelial cell proliferation (**Supplementary Figure 1**). We then performed GO analysis of these differentially expressed genes and found that the functions of these dysregulated genes were involved in several important cell processes, including cell division, proliferation, and nucleus division (**Figure 1C**).

We hypothesized that the HUVEC proliferation repression induced by HDACi treatment is dose- and time-dependent. From treatment of HUVECs with a dose gradient, the HUVEC proliferation rate was decreased by 76% upon treatment with 500 nM TSA (**Figures 1D, E**). From treatment of HUVECs over a time gradient, HUVEC proliferation was decreased by 86% after 24 h of TSA treatment (**Figures 1F, G**). We concluded that treatment with 500 nM TSA for 24 h repressed HUVEC proliferation significantly, and this concentration and time are consistent with those used to obtain the data in GSE5856. These data indicate that HDACi treatment inhibited HUVEC proliferation.

### TSA Reduced the Expression of Proliferation-Related Genes *BIRC5*, *CKS1B*, and *NDC80* in HUVECs

**Figure 2:**
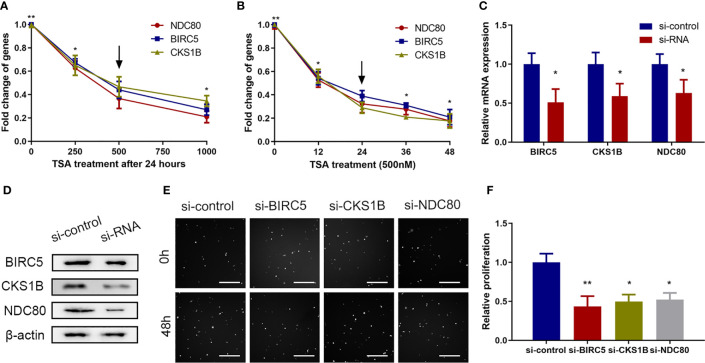
Proliferation-related genes including *BIRC5*, *CKS1B*, and *NDC80* were involved in the effects of TSA on HUVECs *in vitro*. **(A, B)** HUVECs were grown and treated with TSA at different doses **(A)** or for various durations of time **(B)** and the mRNAs of *BIRC5*, *CKS1B*, and *NDC80* were analyzed. **(C)** HUVECs were transfected with siRNAs for 48 h, and knockdown efficacy of the indicated siRNAs was tested with qRT-PCR. **(D)** HUVECs were transfected with siRNA for 48 h and protein levels were assessed by Western blotting assay. **(E)** HUVECs were transfected with the indicated siRNAs for 48 h and the cell viability was examined with Cellomics ArrayScan VTI assay. Scale bar 400 μm. **(F)** Quantification of the data from **(E)**. *P < 0.05, **P < 0.01 *vs*. the control group.

Previous studies have shown that *BIRC5, CKS1B*, and *NDC80* are involved in cell proliferation in a variety of cell types (16–18). Indeed, the analysis of microarray data in this study also identified *BIRC5*, *CKS1B*, and *NDC80* as differentially expressed genes in TSA-treated HUVECs (**Supplementary Figure 2**). We then performed qRT-PCR and found that treatment of HUVECs with 500 nM TSA for 24 h significantly reduced the expression of these three genes (**Figures 2A, B**).

Furthermore, we knocked down the expression of *BIRC5*, *CKS1B*, and *NDC80* in HUVECs using siRNAs, along with control siRNA (**Figures 2C, D**), and found that the knockdown of endogenous *BIRC5*, *CKS1B*, and *NDC80* expression reduced HUVEC viability (**Figures 2E, F**). These data showed that reduced *BIRC5*, *CKS1B*, and *NDC80* expressions were involved in TSA-induced viability suppression of HUVECs.

### TSA Suppressed Cell Proliferation of HUVECs Through Downregulating STAT5A

**Figure 3:**
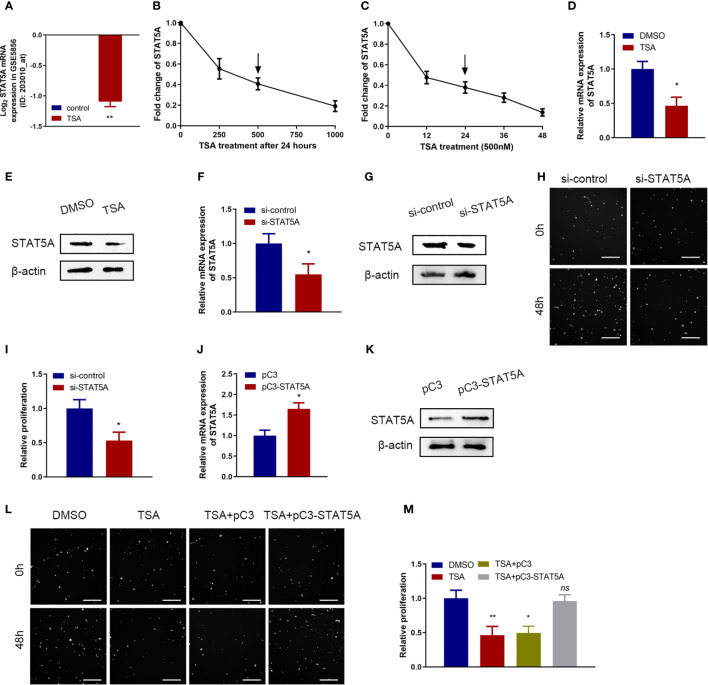
Roles of signal transducer and activator of transcription 5 A (STAT5A) in cell proliferation inhibition of TSA on HUVECs. **(A)** Fold change of STAT5A expression in the microarray data (#GSE5856). **(B, C)** HUVECs were incubated with different doses of TSA **(B)** or for various durations of time **(C)**. mRNA expression of STAT5A was tested by qRT-PCR. **(D, E)** STAT5A mRNA level **(D)** and protein level **(E)** at 48 h after 500 nM TSA treatment were measured by qRT-PCR and Western blotting assay. **(F, G)** Knockdown efficacy of siRNA transfection into HUVECs was analyzed by qRT-PCR **(F)** and Western blotting assay **(G)**. **(H)** HUVECs were transfected with si-STAT5A or si-control for 48 (h) Cell proliferation was examined with Cellomics ArrayScan VTI assay. Scale bar 400 μm. **(I)** Quantification of the data from **(H)**. **(J, K)** HUVECs were transfected with STAT5A plasmid (pC3-STAT5A) or empty plasmid (pC3) and then subjected to qRT-PCR analysis **(J)** and Western blot analysis **(K)**. **(L)** HUVECs were treated with DMSO, TSA, TSA+pC3, or TSA+pC3-STAT5A, and cell proliferation was analyzed by Cellomics ArrayScan VTI assay. Scale bar 400 μm. **(M)** Quantification of the data from **(L)**. *P < 0.05, **P < 0.01 *vs*. the control group. ns, not significant.

STAT5A protein is an important transcriptional activator to maintain expression of a great deal of genes in various biological processes including cell proliferation (19). So, we wanted to know whether STAT5A is involved in TSA-induced cell proliferation inhibition of HUVECs. In the microarray data, TSA significantly downregulated STAT5A expression (**Figure 3A**). Further qRT-PCR assay revealed that TSA treatment inhibited the level of STAT5A mRNA in a dose- and time-dependent manner (**Figures 3B, C**). Moreover, after treatment of HUVECs with 500 nM TSA for 24 h, the levels of STAT5A mRNA and protein were significantly lower than those in cells with control treatment (**Figures 3D, E**). These results suggested that STAT5A may contribute to TSA effects in HUVECs.

Next, we explored the functions of STAT5A in HUVECs by knocking down STAT5A expression using STAT5A siRNAs. qRT-PCR and Western blotting assay confirmed the knockdown effect of siRNA-STAT5A in HUVECs (**Figures 3F, G**). When STAT5A was knocked down, we observed a significant decrease of cell proliferation in HUVECs (**Figures 3H, I**). Thus, knockdown of STAT5A expression *in vitro* inhibited HUVEC proliferation. Then, we designed a rescue experiment to investigate whether STAT5A mediated TSA effects in cell proliferation of HUVECs. qRT-PCR and Western blotting assay confirmed the overexpression effect of STAT5A vector in HUVECs (**Figures 3J, K**). In cell proliferation assay, STAT5A expression could partially rescue the TSA-induced cell proliferation inhibition of HUVECs (**Figures 3L, M**). These results demonstrated that TSA suppressed the cell proliferation of HUVECs *via* downregulating STAT5A expression.

### STAT5A Mediated the Transcription of *BIRC5*, *CKS1B*, and *NDC80*

**Figure 4:**
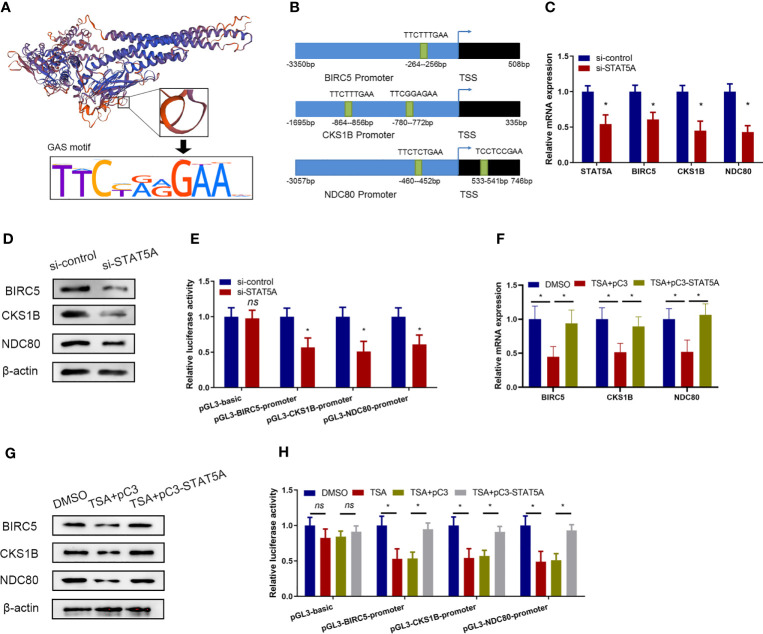
TSA suppressed *BIRC5*, *CKS1B*, and *NDC80* transcription *via* STAT5A. **(A)** Schematic representation of DNA binding motif of STAT5A. **(B)** Predicted binding sites from *BIRC5*, *CKS1B*, and *NDC80* promoter regions for STAT5A. **(C, D)** HUVECs were transfected with si-control or si-STAT5A. *BIRC5*, *CKS1B*, and *NDC80* expressions were assessed by qRT-PCR **(C)** and Western blotting assay **(D)**. **(E)** *BIRC5*, *CKS1B*, and *NDC80* promoter plasmids [regions: −3,350 to 508 (*BIRC5*), −1,695 to 335 (*CKS1B*), and −3,057 to 746 (*NDC80*)] were co-transfected with si-STAT5A or si-control into HUVECs. HUVECs were then subjected to luciferase assays. **(F, G)** HUVECs were treated with DMSO or TSA, transfected with pC3 or pC3-STAT5A, and then were subjected to qRT-PCR analysis **(F)** and Western blotting analysis **(G)** to examine *BIRC5*, *CKS1B*, and *NDC80* expressions. **(H)** HUVECs were transfected with different *BIRC5*, *CKS1B*, and *NDC80* promoter plasmids, treated with DMSO or TSA, transfected with pC3 or pC3-STAT5A, and subjected to luciferase assays. *P < 0.05 *vs.* the control group. ns, not significant.

A previous study demonstrated that the STAT5A protein could recognize the target genes *via* GAS-binding sites and activated gene transcription (20). The interaction between STAT5A protein and the target genes through GAS-binding sites (TTCNNNGAA/AAGNNNCTT) is displayed in **Figure 4A**. We scanned the *BIRC5*, *CKS1B*, and *NDC80* promoter regions and observed several imperfect STAT5A-binding sites (**Figure 4B**). So, we hypothesized that STAT5A mediated the transcription of *BIRC5*, *CKS1B*, and *NDC80.* qRT-PCR and Western blotting assay demonstrated that the knockdown of STAT5A downregulated *BIRC5*, *CKS1B*, and *NDC80* expression in HUVECs (**Figures 4C, D**). Then, we constructed different plasmids carrying the *BIRC5*, *CKS1B*, and *NDC80* promoters and performed a luciferase reporter assay in HUVECs. As shown in **Figure 4E**, knockdown of STAT5A significantly reduced the firefly/Renilla luciferase activity of *BIRC5*, *CKS1B*, and *NDC80* promoters compared with the control siRNA. These results suggested that STAT5A played important roles in maintaining the transcription of *BIRC5*, *CKS1B*, and *NDC80.*

Furthermore, we investigated whether TSA treatments involved STAT5A-mediated transcription of *BIRC5*, *CKS1B*, and *NDC80* in HUVECs. We treated the HUVECs with TSA and transfected the STAT5A overexpression vector simultaneously. Compared with the control treatments, STAT5A overexpression partially rescues *BIRC5*, *CKS1B*, and *NDC80* expressions which were downregulated by TSA in HUVECs (**Figures 4F, G**). This suggested that STAT5A mediated TSA-induced downregulation of *BIRC5*, *CKS1B*, and *NDC80*. Furthermore, the luciferase reporter assay confirmed that TSA treatments reduced the firefly/Renilla luciferase activity of *BIRC5*, *CKS1B*, and *NDC80* promoters. However, STAT5A overexpression blocked the reduction effect of TSA on the firefly/Renilla luciferase activity of *BIRC5*, *CKS1B*, and *NDC80* promoters (**Figure 4H**). These results revealed that TSA downregulates *BIRC5*, *CKS1B*, and *NDC80* transcription through STAT5A repression.

### STAT5A Directly Interacted With the Promoter of *BIRC5*, *CKS1B*, and *NDC80 via* GAS Sites

**Figure 5:**
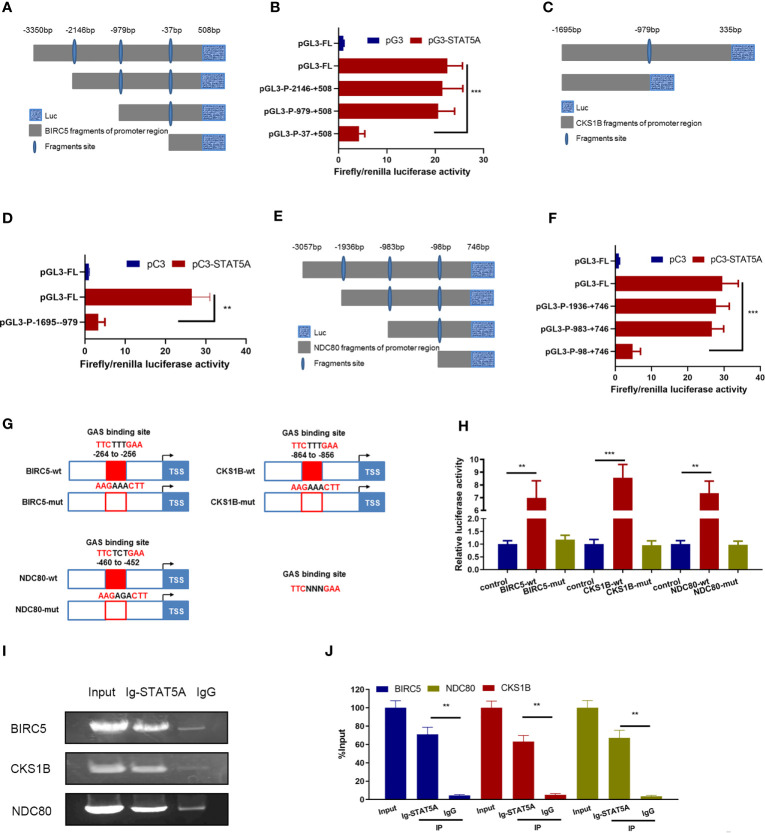
STAT5A directly bound to *BIRC5*, *CKS1B*, and *NDC80* promoters *via* GAS sites. **(A)** Schematic diagram of *BIRC5* promoter reporter plasmids. **(B)** HUVECs received the indicated treatments including pC3, pC3-STAT5A plasmids, and TSA for 48 (h) HUVECs were subjected to luciferase assays. **(C)** Schematic truncations of *CKS1B* promoter reporter plasmids. **(D)** HUVECs received the indicated treatments including pC3, pC3-STAT5A plasmids, and TSA for 48 (h) HUVECs were subjected to luciferase assays. **(E)** Schematic truncations of *NDC80* promoter reporter plasmids. **(F)** HUVECs were treated with TSA and *NDC80* promoter truncations, pC3, or pC3-STAT5A and subjected to luciferase assays. **(G)** Schematic illustration of wild type and mutants for STAT5A binding to *BIRC5*, *CKS1B*, and *NDC80* promoter. **(H)** HUVECs were transfected with different plasmids for 48 h and subjected to luciferase reporter assay. **(I, J)** ChIP assay with an anti-STAT5A or IgG antibody was performed to detect the interaction of STAT5A and *BIRC5*, *CKS1B*, and *NDC80* promoter. Graph of agarose gel electrophoresis for the indicated PCR products **(I)**. **(J)** Enrichment of promoter regions for STAT5A was tested b*y* qRT-PCR. FL, full length. **P < 0.01, ***P < 0.001 *vs.* the control group.

The data described above demonstrated that STAT5A mediated the transcription repression of proliferation-related genes including *BIRC5*, *CKS1B*, and *NDC80* under TSA treatment. We next assessed whether STAT5A directly interacted with the promoters of *BIRC5*, *CKS1B*, and *NDC80*. We constructed luciferase reporter vectors containing different truncations of the *BIRC5* promoter region (**Figure 5A**). Then, the responding luciferase vectors containing different fragments of *BIRC5* promoter were co-transfected with STAT5A overexpression vector into the HUVECs under TSA treatment. As shown in **Figure 5B**, STAT5A activated the luciferase reporter activity of the full length of the *BIRC5* promoter. However, when the −979- to −37-bp fragment of the *BIRC5* promoter was deleted, STAT5A failed to upregulate the luciferase reporter activity. This suggested that the −979- to −37-bp fragment of the *BIRC5* promoter may contain the binding sites of STAT5A. Luciferase reporter vectors containing different truncated *CKS1B* promoters are displayed in **Figure 5C**. The -1695 bp to -979-bp fragment of the CKS1B promoter didn’t result in STAT5A-induced transcription of the luciferase reporter gene (**Figure 5D**). This suggested that activation of CKS1B promoter by STAT5A was mainly dependent on the remaining fragment of CKS1B promoter, the -979 bp to 335-bp. Additionally, we also cloned different truncations of NDC80 promoter in to the luciferase reporter vector (**Figure 5E**). The dual luciferase reporter gene assay showed that STAT5A activated NDC80 through -983 bp to -98-bp fragment of the BIRC5 promoter (**Figure 5F**).

Next, we wanted to identify the direct binding sites of *BIRC5*, *CKS1B*, and *NDC80* promoters for STAT5A interaction. The potential GAS sites from the responsible promoter regions identified above were scanned by sequence alignment and displayed (**Figure 5G**). Plasmids carrying the wild-type and mutated GAS sites of these three gene promoters were constructed and co-transfected with STAT5A into HUVECs to perform dual luciferase reporter gene assay (**Figure 5G**). Compared to the wild-type promoters of BIRC5, *CKS1B*, and *NDC80* mutants of the predicted GAS sites in their promoter abolished the STAT5A-induced upregulation of the luciferase activity (**Figure 5H**). This indicated that the GAS sites including −264 to −256 bp of the *BIRC5* promoter, −864 to −856 bp of the *CKS1B* promoter, and −460 to −452 bp of the *NDC80* promoter were responsible for STAT5A-activated transcription. Finally, we performed the ChIP assay to confirm the direct DNA–protein interaction between STAT5A and *BIRC5*, *CKS1B*, and *NDC80* promoters. The ChIP data revealed that IgG did not precipitate the identified promoter regions of *BIRC5*, *CKS1B*, and *NDC80*, whereas the antibody against STAT5A precipitated the identified promoter regions of *BIRC5*, *CKS1B*, and *NDC80* (**Figures 5I, J**). This supported the hypothesis that STAT5A activated the transcription of *BIRC5*, *CKS1B*, and *NDC80* by directly binding their promoter region. All of these results revealed that STAT5A directly bound to promoters of *BIRC5*, *CKS1B*, and *NDC80 via* GAS sites and activated their transcription.

### TSA Suppressed the Tube Formation of Cancer-Derived Endothelial Cells *via* Downregulation of STAT5A

**Figure 6:**
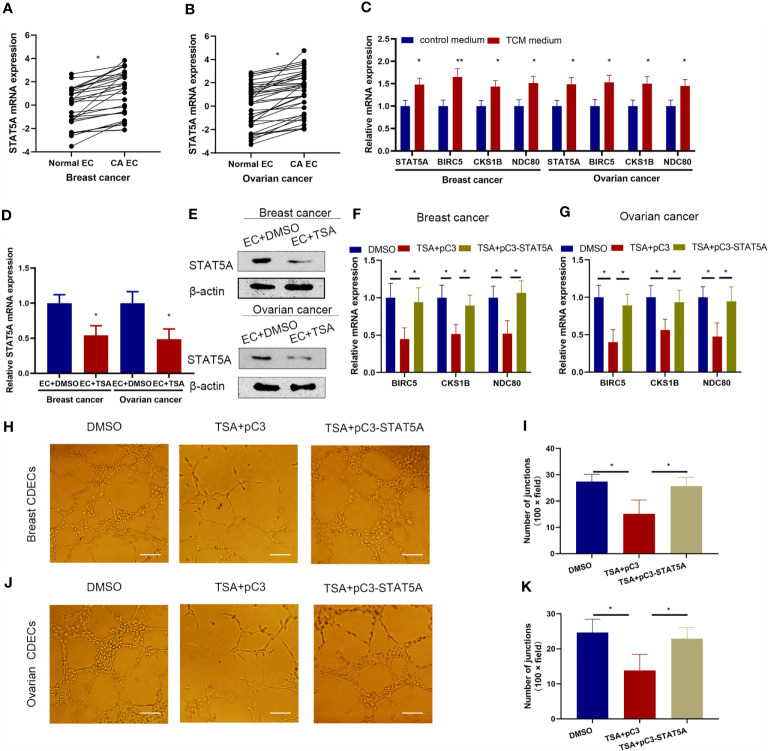
STAT5A also mediated TSA-induced inhibition of tube formation in the cancer angiogenesis model. **(A, B)** Human normal endothelial cells, breast **(A)**, and ovarian CDECs **(B)** were isolated and subjected to qRT-PCR to test the mRNA expression of STAT5A. HUVECs were treated in control medium or breast and ovarian TCM and subjected to qRT-PCR. **(C)** HUVECs were cultured in the control medium or TCM from human breast and ovarian cancer cells. Expression levels of STAT5A, *BIRC5*, *CKS1B*, and *NDC80* were tested by qRT-PCR. **(D, E)** Human breast and ovarian CDECs were treated with DMSO and TSA. Expression of STAT5A was examined by qRT-PCR **(D)** and Western blotting assay **(E)**. **(F, G)** Human breast **(F)** and ovarian CDECs were treated with DMSO, TSA, pC3, and pC3-STAT5A. Expression levels of *BIRC5*, *CKS1B*, and *NDC80* were measured by qRT-PCR. **(H, J)** Breast and ovarian CDECs were treated with TSA and the indicated vectors. Representative graphs of tube formation for breast **(H)** and ovarian **(J)** CDECs were shown. **(I, K)** Quantification data for *in vitro* tube formation of breast **(I)** and ovarian **(K)**. Scale bar 100 μm. Three experiments were replicated. *P < 0.05, **P < 0.01 *vs*. the control group.

The expression level of STAT5A was first quantitatively analyzed in a panel of human cancer-derived endothelial cells (CDECs) including breast and ovarian CDECs. The results showed that STAT5A expression was increased in both breast and ovarian CDECs compared with its expression levels in adjacent normal endothelial cells (**Figures 6A, B**). Furthermore, when HUVECs were treated with breast and ovarian cell-derived TCM, STAT5A, *BIRC5*, *CKS1B*, and *NDC80* expressions were all increased (**Figure 6C**). These results support the notion that STAT5A and its downstream targets may play important roles in cancer angiogenesis. Next, we wanted to know whether TSA displayed the same effects on gene regulation in breast and ovarian CDECs. qRT-PCR and Western blotting assay showed that TSA decreased mRNA and protein levels of STAT5A in both breast and ovarian CDECs (**Figures 6D, E**). Furthermore, TSA also decreased the expression of STAT5A downstream targets including *BIRC5*, *CKS1B*, and *NDC80* at the mRNA level, but STAT5A overexpression disrupted the suppression of *BIRC5*, *CKS1B*, and *NDC80* expression induced by TSA in both breast and ovarian CDECs (**Figures 6F, G**). These results suggested that TSA-associated STAT5A-mediated transcription of *BIRC5*, *CKS1B*, and *NDC80* also existed in CDECs. Finally, in the tube formation model, TSA reduced tube branch formation by the breast and ovarian CDECs cultured in TCM, while STAT5A overexpression rescued branch formation by TSA-treated breast and ovarian CDECs (**Figures 6H–K**). All of these results suggested that TSA also inhibited *BIRC5*, *CKS1B*, and *NDC80* expression and angiogenesis *via* STAT5A in the CDEC angiogenesis model.

## Discussion

**Figure 7:**
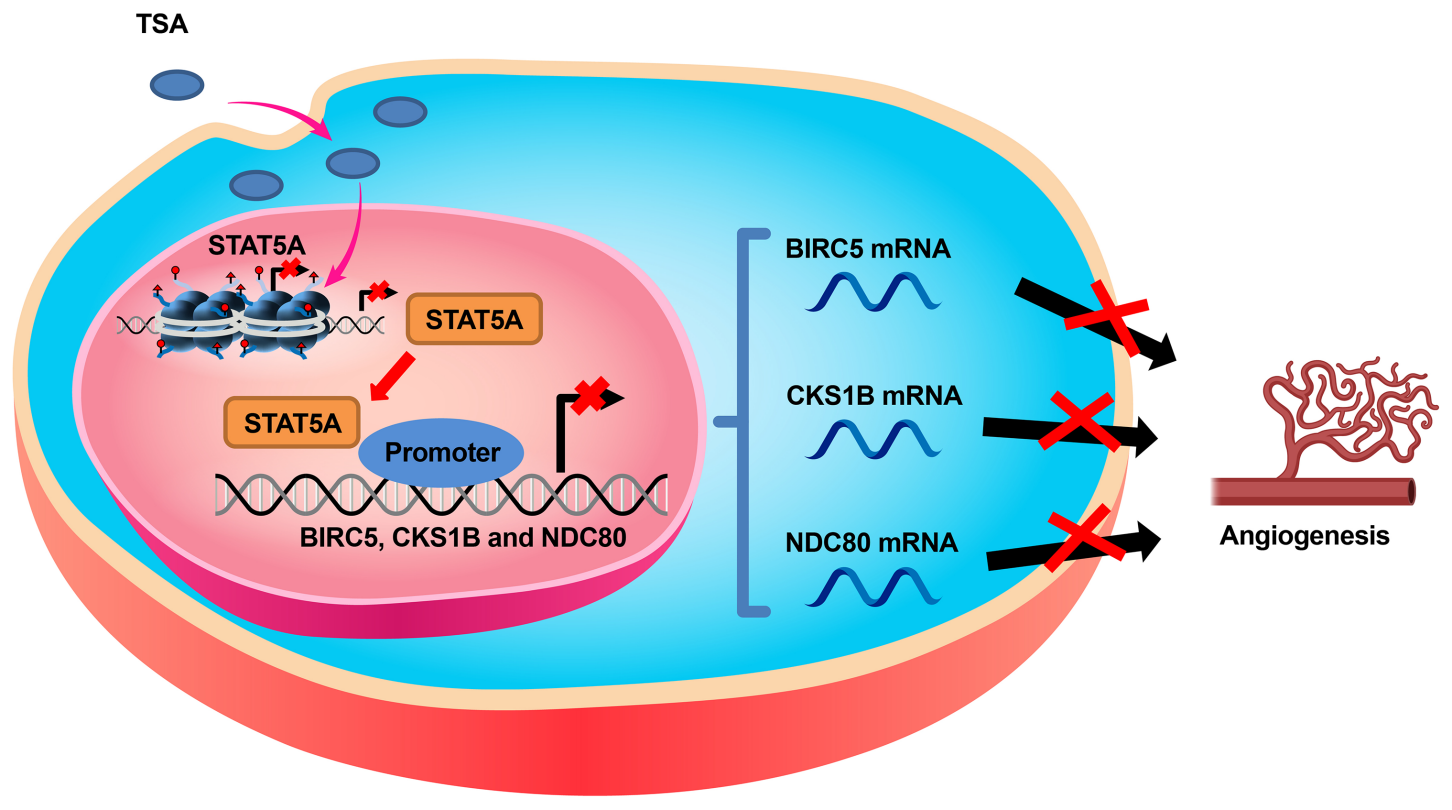
Schematic diagram of the mechanism by which TSA inhibits the expression of *BIRC5*, *CKS1B*, and *NDC80* through suppressing STAT5A, resulting in angiogenesis suppression of endothelial cells.

Clinical studies have demonstrated that HDACi can affect different biological processes (21). In the present study, we explored the direct effects of TSA on endothelial cell gene expression, proliferation, and tube formation. Our main findings were as follows: 1) TSA reduced HUVEC proliferation by STAT5A-mediated downregulation of proliferation-related genes including *BIRC5*, *CKS1B*, and *NDC80*; 2) STAT5A directly bound to those gene promoters and activated their transcription *via* special sequences named GAS sites; and 3) TSA also suppressed tube formation *via* the STAT5A–*BIRC5*, *CKS1B*, and *NDC80* axis in CDEC models (**Figure 7**). This new insight into the mechanisms of action of TSA in endothelial cells could support the development of novel strategies for future combination therapies for treating angiogenesis-associated diseases.

Deregulated angiogenesis plays a key role in various diseases. TSA, as an HDACi, was found to play conflicting roles in different disease models. In this study, we observed that TSA inhibited the proliferation of HUVECs, which supported the notion that TSA can directly suppress angiogenesis of endothelial cells. The opposite effect of TSA on angiogenesis in other diseases may depend on its indirect effects on the microenvironment. Different HDACi have been shown to suppress different signaling pathways and the corresponding downstream genes (22). The results of the present study showed that BIRC5, CKS1B, and NDC80 are involved in TSA-induced inhibition of endothelial cell proliferation. *BIRC5* is a member of the inhibitor of apoptosis (IAP) gene family, and IAP genes encode negative regulatory proteins that prevent apoptotic cell death (23). Specifically, *BIRC5* is overexpressed in different human cancers as an oncogene and, thus, is a key target for anticancer therapy (16). *CKS1B* binds to the catalytic subunit of cyclin-dependent kinases and is involved in the control of their biological functions, and inhibition of *CKS1B* expression blocks breast cancer cell entry into the M phase of the cell cycle (17). *NDC80* is one of the proteins of the outer kinetochore and is involved in the regulation of cell mitosis (18), giving it an obvious role in cell proliferation. We found that *BIRC5*, *CKS1B*, and *NDC80* are key proliferation-related genes and their expression is reduced by TSA in HUVECs.

The results of the present study may raise a question regarding how these pro-proliferation-related genes are involved in endothelial cell proliferation. Previous studies have reported that *BIRC5*, *CKS1B*, and *NDC80* expression is regulated at the transcriptional level, as promoters of these genes contain specific sites for the activation of transcription (16–18, 24). The STAT5 gene, which encodes one of the seven members of the STAT family of proteins, was originally identified as a novel member of the cytokine-regulated transcription factor gene family and involved in prolactin-induced gene expression (25). Activated STAT5 can bind to the DNA sequence 5′-TTCNNNGAA-3′, also known as the GAS, to activate the transcription of target genes (26). STAT5 expression was found to regulate hematopoietic cell proliferation, differentiation, and survival (27). Notably, aberrant STAT5 expression occurs in a wide range of human cancers (28). A previous study demonstrated that STAT5 activation is the key event in the generation of endothelial morphogenesis and angiogenesis, and another study showed that activated STAT5 can regulate cell proliferation, differentiation, and survival in a variety of cells (29). Indeed, our present study revealed that activated STAT5A protein could bind to the promoters of the *BIRC5*, *CKS1B*, and *NDC80* genes to activate their transcription. In addition, TSA was found to inhibit tube formation by CDECs *via* STAT5A-mediated transcription suppression of *BIRC5*, *CKS1B*, and *NDC80* expression. Further research is needed to elucidate how TSA treatment inhibits STAT5A expression in HUVECs. Acetylation modification has been demonstrated not only to regulate transcription of STATs by histone acetylation but also to regulate protein stability and dimer formation of STATs *via* residue acetylation, all of which were critical for transcription activity of STAT proteins. Considering that both the mRNA and the protein levels of STAT5A were downregulated by TSA, we concluded that TSA mainly suppressed STAT5A expression *via* histone acetylation. However, further study is needed to investigate whether TSA affected STAT5A function through residue acetylation in HUVECs (30).

In conclusion, the present study provides the first demonstration that TSA directly inhibits the proliferation of endothelial cells *via* STAT5A-mediated transcription repression of proliferation-related genes including *BIRC5*, *CKS1B*, and *NDC80*. Additionally, the TSA–STAT5A–*BIRC5*, *CKS1B*, and *NDC80* axis also worked in cancer cell angiogenesis models. Importantly, this study provides proof-of-principle evidence, and much research is still needed to investigate and confirm the effects of TSA, or other HDACi, on endothelial cell activities.

## Data Availability Statement

The raw data supporting the conclusions of this article will be made available by the authors, without undue reservation.

## Ethics Statement

This study was registered and approved by the Ethics Committee of the First Affiliated Hospital of Fourth Military Medical University (registration number: KY20213431-1). The patients/participants provided their written informed consent to participate in this study.

## Author Contributions

WL and WB conceived and designed the study. YL, YZ, HP, LB, and LW performed the *in vitro* analysis. JZ and LB analyzed the data and prepared the figures. HZ, WB, and WL wrote the manuscript. All authors contributed to the article and approved the submitted version.

## Funding

This study was supported by the National Natural Science Foundation of China (81702554 to YL, 81802661 to WB).

## Conflict of Interest

The authors declare that the research was conducted in the absence of any commercial or financial relationships that could be construed as a potential conflict of interest.

## Publisher’s Note

All claims expressed in this article are solely those of the authors and do not necessarily represent those of their affiliated organizations, or those of the publisher, the editors and the reviewers. Any product that may be evaluated in this article, or claim that may be made by its manufacturer, is not guaranteed or endorsed by the publisher.
