# Supplementary material for: Histone Deacetylase Inhibitor Trichostatin A Reduces Endothelial Cell Proliferation by Suppressing STAT5A-Related Gene Transcription
Source: Front Oncol. 2021 Sep 23;11:746266. doi: 10.3389/fonc.2021.746266 (PMC8506210; doi:10.3389/fonc.2021.746266)
Supplement: Supplementary file 1 [file DataSheet_1.docx]

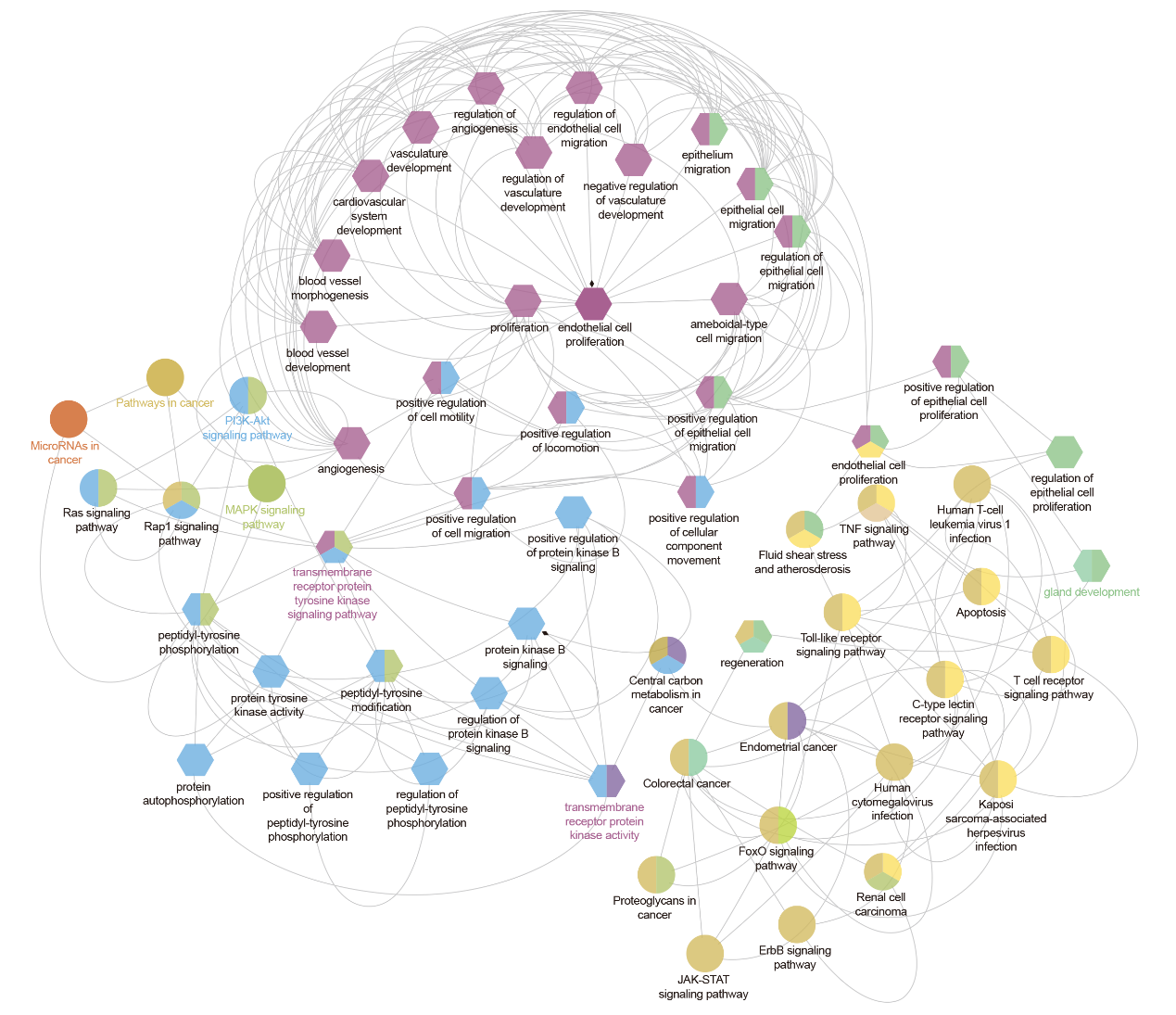


**FIGURE S1 |** Pathway enrichment and interactions between pathways were accomplished using the ClueGO and CluePedia plugins of Cytoscape software. Cluster analysis by ClueGO and CluePedia plugins showed enrichment of meaningful pathways based on statistical analysis, with no duplication between them. Cluster one mainly includes endothelial cell proliferation and the related signaling pathways.


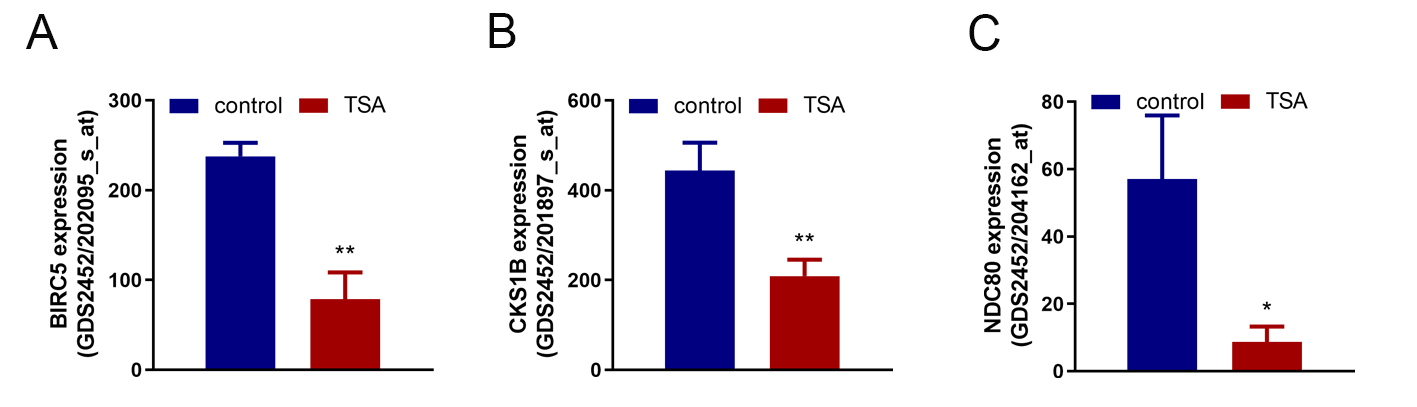


**FIGURE S2 |** Microarray data. *BIRC5* **(A)**, *CKS1B* **(B)** and *NDC80* **(C)** mRNA levels were determined from the microarray data in the GEO database (GSE5856) with RNA probes. **P* < 0.05, ***P* <0.01 vs. the control group.
